# Racism and psychosis: an umbrella review and qualitative analysis of the mental health consequences of racism

**DOI:** 10.1007/s00406-022-01468-8

**Published:** 2022-08-24

**Authors:** Felicia Boma Lazaridou, Saskia J. Schubert, Tobias Ringeisen, Jakob Kaminski, Andreas Heinz, Ulrike Kluge

**Affiliations:** 1 Department of Psychiatry and Psychotherapy, Charité University Medicine, Campus Mitte, Berlin, Germany; 2 National Discrimination and Racism Monitor, German Institute for Integration and Migration Research - DeZIM, Berlin, Germany; 3 Department of Migration, Mental and Physical Health and Health Promotion, Berlin Institute of Integration and Migration Research (BIM), Humboldt University, Berlin, Germany; 4 Berlin School of Economics and Law, Berlin, Germany; 5 Department of Psychiatry and Psychotherapy, Charité University Medicine, Alexianer St. Hedwig-Hospital, Berlin, Germany

**Keywords:** Psychosis, Racism, Meta-analysis, Umbrella review, Mixed methods research

## Abstract

Black people and People of Color are disproportionately affected by racism and show increased rates of psychosis. To examine whether racialized migrant groups are particularly exposed to racism and therefore have higher risks for psychosis, this paper (1) systematically assesses rates of psychosis among racialized migrant groups concerning the country of origin, and (2) analyzes interviews regarding the association of racism experiences with psychosis-related symptoms in racialized Black people and People of Color populations in Germany. We present an umbrella review of meta-analyses that report the incidence of positive symptoms (e.g., hallucinations and delusions) and negative symptoms (e.g., apathy and incoherent speech) of diagnosed schizophrenia, other non-affective psychotic disorders (e.g., schizoaffective disorder) or first-episode psychosis among migrants by country of origin. We also report 20 interviews with first- and second-generation migrants racialized as Black and of Color in Germany to capture and classify their experiences of racism as well as racism-associated mental health challenges. In the umbrella review, psychosis risk was greatest when migration occurred from developing countries. Effect size estimates were even larger among Caribbean and African migrants. In the qualitative study, the application of the constant comparative method yielded four subordinate themes that form a subclinical psychosis symptomatology profile related to experiences of racism: (1) a sense of differentness, (2) negative self-awareness, (3) paranoid ideation regarding general persecution, and (4) self-questioning and self-esteem instability. We here provide converging evidence from a quantitative and qualitative analysis that the risk of poor mental health and psychotic experiences is related to racism associated with minority status and migration.

**Supplementary Information:**

The online version contains supplementary material available at 10.1007/s00406-022-01468-8.

## Introduction

Psychosis is more prevalent among migrants than among non-migrants [1, 2]. A systematic review and meta-analysis of the influence of gender, urbanicity, immigration and socio-economics on psychosis, found an incidence rate of 3.09 (95% CI, 2.74—3.49) in migrants compared to non-migrants [3]. Certain forms of migration, such as forced migration, carry a greater risk of psychosis due to the confluence of stress and potential traumatization [1]. Stressful life events directly preceding onset, mediated by genetic vulnerability, are implicated in the etiology of psychosis in adolescents and adult populations [4]. Migrants are more exposed than non-migrants to area risk factors like population density, social fragmentation and deprivation, and social risk factors [5]. Evidence suggests that racism is an important social risk factor for the severity of psychotic symptoms among first and second-generation migrants (*r* = 0.264, *p* = 0.005) [6]. Furthermore, migrants with darker skin complexion are more likely to develop psychosis than migrants with lighter skin complexion (RR = 4.19, CI 3.42—5.14) [2]. This finding is generally consistent with the literature on involuntary admissions. A Canadian study, for example, reported an elevated risk among African migrants (RR = 1.24, 95% CI 1.04–1.48) and Caribbean migrants (RR = 1.29, 95% CI 1.07–1.56) compared to European migrants [7].

The fact that such effects are mediated by social risk factors, such as racism, is suggested by findings pertaining to area risk factors such as “ethnic density”, with higher psychosis rates among Black people and People of Color when they live in less diverse neighborhoods and experience higher rates of social discrimination [8, 9]. Psychosocial stress symptoms are associated with neural correlates of emotionality, such as the pain of social loss and rejection [10] and share neural substrates with physiological stress symptoms [11]. The stress vulnerability model [12] connects two conceptual frameworks: (1) the weathering concept [13, 14] refers to the disproportionate and cumulative health consequences of racism and discrimination, such as prenatal complications and mortality; and (2) the concept of allostatic load [15] refers to the imprint of racism on mind and brain via chronic physiological activation patterns, such as blood pressure and cortisol levels. Both postulate that the effects of stress on the brain at the cellular level are the mechanisms through which interpersonal racism (individualized attacks), institutional racism (policies and procedures), and structural racism (historical practices and societal reality) can lead to various adverse mental health outcomes, including psychosis [9, 16, 17].

An area of research known as the ‘ultra-high-risk state for psychosis paradigm’ [18] predicts the risk of conversion from basic and attenuated symptomatology (which are common subclinical psychosis-like signs), to clinical psychosis [19, 20]. Exposure to racism is positively associated with the distribution of subclinical psychosis symptomatology in non-clinical populations [21]. This paradigm is important in terms of early intervention, but it is not without controversy (see [22] for a review of the pragmatic dangers). A meta-analysis by Paradies et al. [23] concludes that racism is a social determinant of health and a review by Fusar-Poli et al. [24] concludes that governmental support of innovations aimed at strengthening the social determinants of health is required. With this is in mind, mental health leaders and their institutions must prioritize professional competence that considers culture, racism and migration-related stress factors in the context of seeking to understand and remedy mental health burden [25]. Evidence suggests that in terms of paranoid thinking, the cognitive evaluation of motives, meaning, and relevance around racism affects the general population and persons with attenuated symptomatology to the same degree [26]. In current biopsychosocial models of indicated risk and prevention, the higher baseline of attenuated symptomatology is the most important risk factor—a risk factor which is magnified by a family history of psychosis [27]. The clinical high-risk paradigm should operate within an indicated risk and prevention framework [28]. A universal risk and prevention framework aims to detect psychosis in certain at-risk groups in the general population such as Caribbean migrants in general or Black men in general. As such, a universalized approach to psychosis undermines the experiences of the individual patient and leads to overdiagnosis in entire groups of people who become essentialized and treated as categorically different, objective “facts”. In contrast, an indicated risk and prevention framework focuses on reducing the current attenuated psychosis symptoms and reducing the functional decline in at-risk individuals who are engaged in help-seeking behaviors for mental health problems [29].

In this paper, we review meta-analytical findings on the variance of psychosis incidence among migrants by country of origin to provide important clues regarding our central thesis, to explain excess psychosis rates in racialized migrant groups. In parallel, we conduct a qualitative study to describe Black people and People of Color's experiences of racism and mental distress. Rather than focusing solely on clinical populations, investigating racism as a risk factor for the onset of subclinical psychosis symptomatology in non-clinical populations may be an effective way to supplement our understanding of the social etiology of psychosis. The qualitative study focuses primarily on the phenomenological form and content in which racism-induced disturbances manifest.

## Methods

### Umbrella review of meta-analyses on psychosis risk related to countries of origin of migrants

#### Search strategy and selection criteria

Individual meta-analyses assessing migration and psychosis were systematically searched for and evaluated. The final search strategy used the keywords (“migration” OR “trauma” OR “discrimination” OR “racism”) and (“psychosis” OR “non-affective psychosis” OR “schizophrenia” OR “first-episode psychosis”) and (“meta-analysis”) with a restriction for publication between January 2018 and December 2020. 139 titles and abstracts were screened. All studies that reported summary risk rates for the incidence of psychotic symptoms or disorders according to the region of origin or arrival or development in the region of origin were included. This screening yielded 18 full-text articles assessed for eligibility. Included peer-reviewed meta-analyses reported (1) pooled relative risk (RR), incident rate ratio (IRR), hazard ratios (HR) or odds ratio (OR) with a 95% confidence interval (CI) or an effect size that was presented in a way that could be converted to the common effect size of Cohen’s d (e.g., Pearson’s correlation coefficient r), (2) the incidence of positive symptoms (e.g., hallucinations and delusions) or negative symptoms (e.g., apathy and incoherence) or diagnosed schizophrenia (SCZ), or other non-affective psychotic disorders (e.g., schizoaffective disorder) or first-episode psychosis. Systematic reviews without meta-analyses were excluded. Articles on drug-induced psychosis were also excluded. The Preferred Reporting Items for Systematic Reviews and Meta-Analyses (PRISMA) guidelines [30] and additional guidance from Fusar-Poli and Radua [31] were followed.

### Data extraction

Three reviewers performed data extraction, and disagreements were resolved in consensus meetings. The different meta-analyses were grouped according to the similarity of the reported factors classifying migrant status. The following countries or regions and their characteristics could be identified as potential candidates for systematic evaluation: (1) categorization of the country according to the *World Economic Situation and Prospects 2020* [32]: “developed”, “developing”; (2) countries/regions of arrival: the United Kingdom (UK), Scandinavia, the Netherlands, Israel, Southern Europe, Canada; (3) countries/regions of origin: the Caribbean, North Africa, Sub-Saharan Africa, the Middle East, New Zealand and Australia, Oceania, Western Europe, Southern and Eastern Europe, the United States (US). Effect size measures (risk ratio: RR, incidence rate ratio: IRR, hazard ratio: HR or Odds-ratio: OR) and corresponding CI of each meta-analysis were extracted. Those measures include population-based incidence studies comparing incidence between migrants and non-migrants. If available, additional heterogeneity measures, such as Cochran's Q and the I^2^ statistic, were extracted. In addition, evidence of publication bias was noted. The extracted data of each meta-analysis are provided in Supplement 1 and included first (FGM) and second-generation migrants (SGM). If separate estimates for those sub-groups were reported, we marked this in the study labels. The methodological quality of included studies was assessed using an adapted version of the validated assessment of multiple systematic reviews (AMSTAR) tool: 1–4 points equal low quality, 5–7 points equal medium quality, and 8–11 points equal high quality [32] (See Supplement 2).

### Statistical analysis

Meta-analysis was performed according to the approach outlined by Harrer et al. [33]. Forest plots were created, allowing a visual comparison of the different countries and their characteristics. All analyses were performed with the {tidyverse} [34], {meta} [35], {metafor} [36], and {dmetar} [36] packages in the statistical software RStudio (see Supplement 3). Extracted effect size estimates were converted to Cohen’s d [37] and used to compare the different studies [33]. A common interpretation categorizes effect sizes in the range of |*d*|< 0.2 as small, |*d*|< 0.5 as moderate, and in the range of |*d*|< 0.8 as large [37]. First, we compared effect sizes concerning the country's development status, and then analysis was grouped for countries of origin and arrival.

### Qualitative interviews regarding experiences of racism and their association with symptoms of psychosis

#### Sample and recruitment

Twenty African migrants (seven men and thirteen women) were interviewed online, in alignment with social distancing guidelines during the COVID-19 pandemic. This study was conducted in accordance with the World Medical Association’s *Declaration of Helsinki* [38]. The ethics board of a major university in Berlin approved it. Participants were recruited through strategically placed posts in two private Facebook groups: one for Black women in Berlin and the other for Black people of all genders in Berlin. For ethical reasons, the posts included the theme of the study (racism, sexism and mental health: a narrative analysis of people of African descent in Germany), as well as the aim of the study plus the possible benefits and risks of participation were outlined. We included information on the themes of the study in the Facebook posts since it was possible that some participants could have found confronting their individual and collective experiences on this topic in an interview somewhat distressing (see Supplement 4). Therefore, they needed to be aware of the potential benefits and risks of participation. This said, it is possible that we recruited mainly people who were already sensitized to the topic of racism—which could be seen as a limitation, but could also be seen as a beneficial contribution to the depth of the research data. Participants who self-identified as of African descent and lived in Berlin were included. One person declined to participate because they felt they had no personal racism experiences in Germany to reflect upon. After stepping out of this person, we recruited another eligible interviewee.

### Data collection

The interview data was collected using a predefined semi-structured topic guide (see Supplement 5). The Black Feminism movement proposed intersectionality as a theory of social identities in the context of converging oppressions and discrimination, as well as the associated disparities and inequities [39]. One central argument of intersectionality is that everyday racism and gendered forms of racism operate simultaneously. It is suggested that research should reflect this interdependence, rather than treating them as independent entities [40]. Therefore, the topic guide was anchored in an interdisciplinary review of the literature and included key areas of inquiry, such as theoretical and experiential perspectives on the connectedness of both racism and sexism to mental health. A total of thirty interview questions were asked. Interviews lasted between 45 and 180 min, four were paused and resumed another day, and all were digitally recorded. The first interview with a woman based in the South of Germany was used as a pilot test and not included in the data analysis process or the final results due to ineligibility because of location. Data saturation was attained after nineteen interviews, therefore twenty interviews in total took place.

### Data analysis

The constant comparative method was used in the analysis [41, 42] in MAXQDA 20.3 (VERBI GmbH). In the deep immersion process, the interviews were listened to and transcribed verbatim by the first author. FBL and SJS then read each transcript repeatedly. At the first coding step, each transcript was fragmented and open-coded, reflecting the meaning of fragments applied in reference to the study aims. Interpretations were discussed, and a consensus was reached. With the formulated hypotheses about patterns and types in mind, FBL and SJS re-immersed themselves in the interpretative coding process to investigate the relationship between themes emerging comparatively across the transcripts. In further consensus meetings, an inventory of superordinate themes was established by building a conceptual profile of the various relationships between codes (see Supplement 6).

## Results

### Umbrella review of meta-analyses on psychosis risk related to countries of origin of migrants

#### Characteristics of the studies

Overall, 519 titles and abstracts were screened. With the resulting search strategy, 139 articles were found and after applying the eligibility criteria 18 meta-analyses were examined in full text. Of these, 5 meta-analyses could be included in the systematic literature review (see Supplement 7). The key characteristics of each included meta-analysis are provided in Table 1.

**Table 1:** Selected characteristics of included meta-analytic studies

Authors	Country	Year	No. of included studies	Location of included studies	Diagnoses	Inclusion period
Bourque, F., van der Ven, E., and Malla, A	Canada	2011	21	United Kingdom (*n* = 9) Netherlands (*n* = 3) Australia (*n* = 1) Sweden (*n* = 3) Denmark (*n* = 2) Israel (*n* = 2) Canada (*n* = 1)	Schizophrenia first episode psychosis psychosis disorders	January 1977—December 2008
Cantor-Graae, E., and Selten, J. P	Sweden	2005	18	United Kingdom (*n* = 12) Australia (*n* = 1) Netherlands (*n* = 3) Sweden (*n* = 1) Denmark (*n* = 1)	Schizophrenia	January 1977—April 2003
Kirkbride, J. B., Errazuriz, A., Croudace, T. J., Morgan, C., Jackson, D., Boydell, J., Murray, R. M., and Jones, P. B	England	2012	83	England	Non-affective psychoses schizophrenia affective psychoses	1950—2009
Nielssen, O., Sara, G., Lim, Y., and Large, M	Australia	2013	n/a	n/a	Psychotic disorders	2001—2010
Selten, J. P., van der Ven, E., and Termorshuizen, F	The Netherlands	2020	48	Europe (*n* = 43) Israel (*n* = 3) Canada (*n* = 2) Australia (*n* = 1)	Non-affective psychoses affective psychoses	January 1977—October 2017

### Summary of meta-analytic associations

In this analysis, “k” refers to the number of studies included in calculating the summary effect size.

**Fig. 1:**
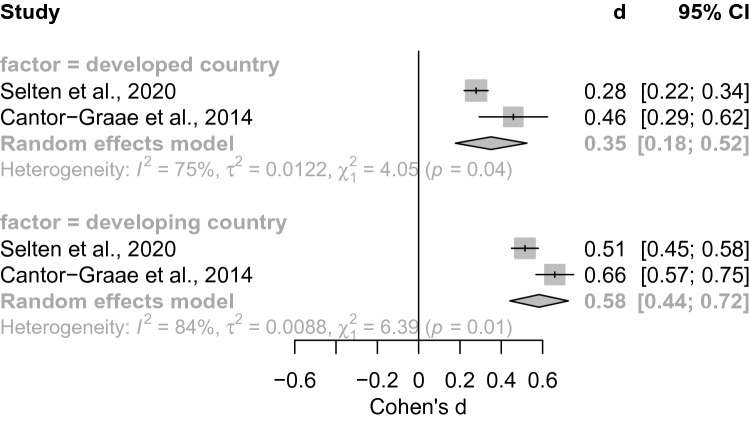
Forest plot of meta-analysis comparing the developmental status of the country of origin. We here show the significant between-group effect depending on the factor developed vs. developing country (*Q* = 4.14, *p* = 0.042). Every meta-analysis estimate corresponds to a gray box. The summary effects are represented by gray diamond shapes. The between-group effect was due to higher estimates (*d* = 0.58, diamond shape) in developing countries as compared to developed countries (*d* = 0.35, diamond shape). We additionally report heterogeneity measures that show significant test statistics (*χ*^2^) for heterogeneity estimates (*I*^2^) and estimates of the between-study variance (*τ*^2^). Abbreviations: *d* = Effect size measure Cohen’s d, CI  confidence interval)

*Development status of country of origin.* As there were more than *k* = 2 studies per subgroup, we conducted subsequent subgroup analysis. We found a significant between-group effect for development status (*Q* = 4.14, *p* = 0.042, see Fig. 1). The random effects model revealed increased risk in developed (*d* = 0.35[0.18; 0.52], *z* = 3.97, *p* < 0.001) and developing countries in mean estimates of psychosis risk (*d* = 0.58[0.44; 0.72], *z* = 8.08, *p* < 0.001).

**Fig. 2:**
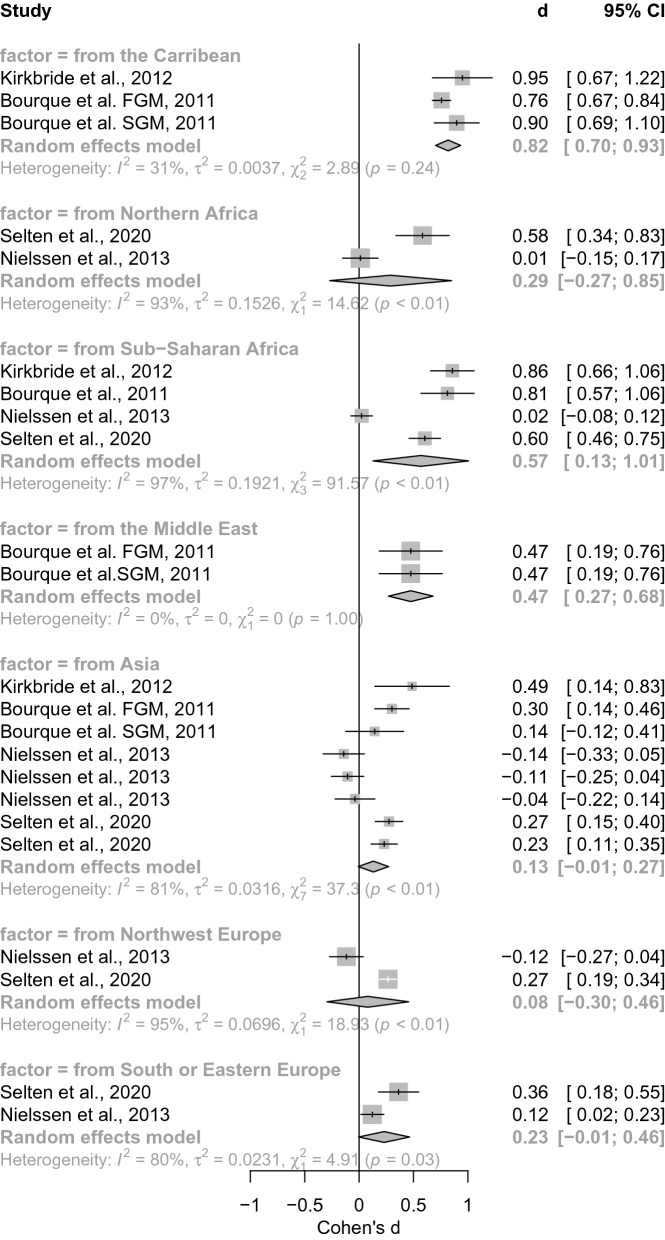
Forest of meta-analysis comparing country of origin We here show the significant between-groups effect depending on the factor from which country migrants were migrating (*Q* = 66.28, *p* < 0.001). Every meta-analysis estimate corresponds to a gray box. The summary effects are represented by gray diamond shapes. We additionally report heterogeneity measures that show significant test statistics (*χ*^2^) for heterogeneity estimates (*I*^2^) and estimates of the between-study variance (*τ*^2^). Abbreviations: *d* = Effect size measure Cohen’s d, CI confidence interval)

*Country of origin.* There were also more than *k* = 2 studies per subgroup, so we calculated subsequent subgroup analysis. We found a significant between-group effect for country of origin (*Q* = 66.28, *p* < 0.001, see Fig. 2).The random effects model revealed increased risk in subjects migrating from the Caribbean (*d* = 0.82[0.7; 0.93], *z* = 13.95, *p* < 0.001), no difference in subjects migrating from North America (*d* = 0.29[− 0.27; 0.85], *z* = 1.01, *p* = 0.311), increased risk for migrants from Sub-Saharan Africa (*d* = 0.57[0.13; 1.01], *z* = 2.54, *p* = 0.011), increased risk for migrants from the Middle East (*d* = 0.47[0.27; 0.68], *z* = 4.55, *p* < 0.001), no significant difference for migrants from Asia (*d* = 0.13[− 0.01; 0.27], *z* = 1.84, *p* = 0.066), and no significant difference for Northwest Europe (*d* = 0.08[− 0.3; 0.46], *z* = 0.42, *p* = 0.675) and South or Eastern Europe (*d* = 0.23[− 0.01; 0.46], *z* = 1.92, *p* = 0.055).

**Fig. 3:**
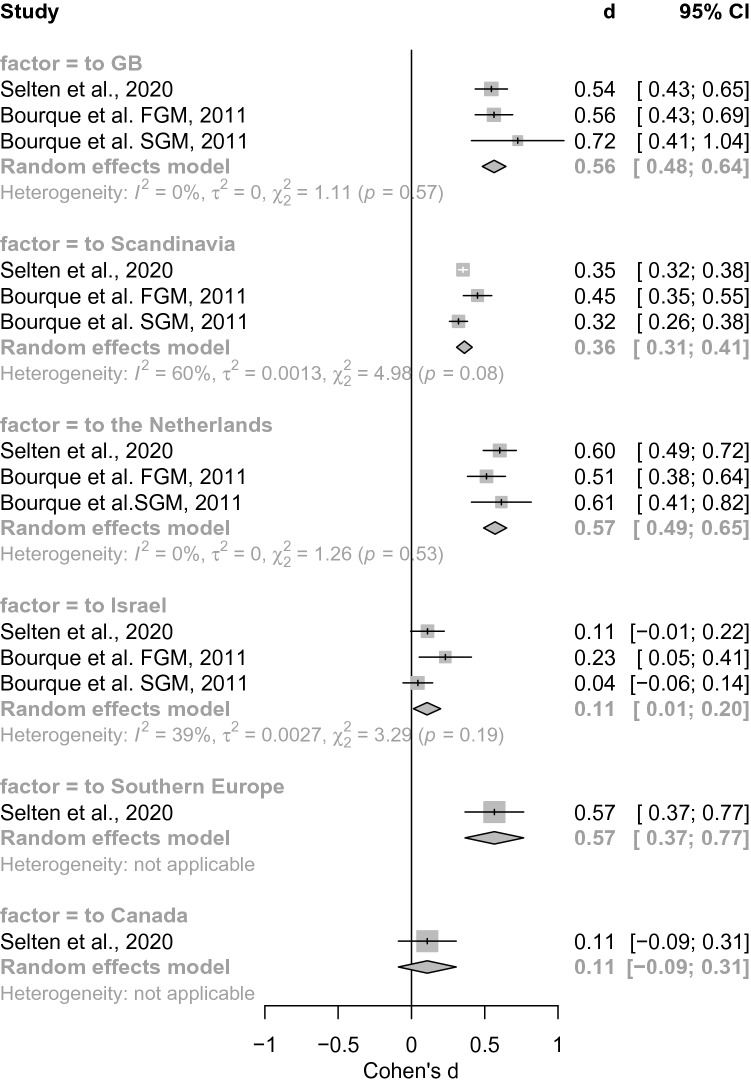
Forest plot of meta-analysis comparing country of arrival. We here show the significant between-group effect depending on the factor to which country migrants were migrating (*Q* = 66.28, *p* < 0.001). Every meta-analysis estimate corresponds to a gray box. The summary effects are represented by gray diamond shapes. We additionally report heterogeneity measures that show significant test statistics (*χ*^2^) for heterogeneity estimates (*I*^2^) and estimates of the between-study variance (*τ*^2^). Abbreviations: *d* = Effect size measure Cohen’s d, CI confidence interval)

*Country of arrival.* There were also more than *k* = 2 studies per subgroup for the majority of sub-groups, depending on country of arrival so we calculated subsequent analysis. We found a significant between-group effect for the country of arrival (*Q* = 85.19, *p* < 0.001, see Fig. 3). The random effects model revealed increased risk in subjects migrating to Great Britain (*d* = 0.56[0.48; 0.64], *z* = 13.69, *p* < 0.001) to Scandinavia (*d* = 0.36[0.31; 0.41], *z* = 13.23, *p* < 0.001) and to the Netherlands (*d* = 0.57[0.49; 0.65], *z* = 14.24, *p* < 0.001), increased risk after migrating to Southern Europe (*d* = 0.57[0.37; 0.77], *z* = 5.54, *p* < 0.001), and no difference was found for subjects migrating to Israel (*d* = 0.11[0.01; 0.2], *z* = 2.24, *p* = 0.025) or to Canada (*d* = 0.11[− 0.09; 0.31], *z* = 1.07, *p* = 0.284).

*Heterogeneity.* The summary results for Caribbean migrants showed moderate heterogeneity, whereas those for sub-Saharan African migrants showed substantial heterogeneity. This is mainly due to Nielssen et al. [43], whose low effect sizes compared to the other studies suggest a possible outlier effect. This effect could also account for the heterogeneity of risk for migrants from the different regions, as Nielssen et al. [43] also consistently reported the smallest effect sizes here.

Moderate effect sizes with low heterogeneity were found for migrants from the Middle East and, apart from in Nielssen et al. [43], for North Africa, Northwest Europe, and Southern and Eastern Europe. In contrast, even without the effect sizes of Nielssen et al. [43], only small effect sizes were found for migrants from Asia, with Kirkbride et al. [44] as a potential outlier reporting effect sizes in the upper-moderate range and having the largest impact on the heterogeneity of this group. If Nielssen et al. [43] are included in the Asian group, there are indications for a possible protective effect of migration from this region, with partly negative effect sizes or confidence intervals whose lower limit is smaller than zero, meaning that there is a lower risk as compared to other migrant groups. The same applies to migrants from Northwest Europe.

In both studies that focused on the developmental status of the country of origin [45, 46], migrants from developing countries, as well as from developed countries, were found to have higher relative risks of developing psychosis compared to the general population. The more recent study by Selten et al. [45] found smaller effect sizes compared to that of Cantor-Graae and Selten [46] where the significant *Q* value for between-group heterogeneity indicates that these differences cannot be explained by variability but are significant. Despite this heterogeneity, both reporting significantly larger effect sizes, for migrants from developing countries, with the relative risk increasing by at least 2.5 times the general population. The number of studies for subgroup analysis included in the calculation was sufficient in the reported cases and consistently above *k* = 2.

Regarding the countries of arrival, the highest values were found for the Netherlands, Great Britain (each with low heterogeneity between the meta-analyses) and Southern Europe, each with effect sizes in the high range. In contrast, effect sizes of medium strength were found for migration to Scandinavia with high heterogeneity. Small effect sizes were found for migration to Israel and Canada at moderate heterogeneity.

### Qualitative interviews regarding experiences of racism and their association with symptoms of psychosis

Interviewees described various subclinical psychosis symptoms that are risk factors for conversion to clinical psychosis. These symptoms can be grouped into four subordinate themes: (1) a sense of differentness, (2) negative self-awareness, (3) paranoid ideation regarding general persecution, and (4) self-questioning and self-esteem instability (see Supplement 6).

### A sense of differentness

Racism causes Black migrants to perceive their bodies as a deviation from the *white* norm, which has negative affective consequences. Interviewees felt that racism constructed boundaries pertaining to ‘normal’ personhood in contrast to ‘different’ personhood. One interviewee said: “When I'm home in Namibia, I'm normal and normal-looking. And I feel normal” (Interviewee 3). Interviewees spoke of a rigid and stereotypical ‘European civilized/African uncivilized’ binary in terms of thought patterns, behavior, speech, as well as physical appearance, which rendered them hyper-visible in public spatial interactions with *white* people. One interviewee said: “I'm always constantly reminded that I'm not a full hundred percent part of the society or not seen as a full part of the society because I'm Black and I have features that don't fall into the racist or stereotypical concept of a German. I'm not *white*” (Interviewee 7). They felt emotionally alienated at the lack of acceptance and the lack of safety in spatial dynamics. Interviewees felt pressure to reconcile the incongruence between their actual self and the forced-ideal self. One interviewee said: “I felt that being an African in Europe, I felt that I had to adjust to come here. I felt the anxiety and the fear that I was feeling was just the process of becoming a much more civilized, African woman.” (Interviewee 9). Many felt a pressure to assimilate to the unpleasant and incorrect expectations of the external *white* gaze that abnormalizes them.

### Negative self-awareness

Racism causes feelings of inadequacy and uncertainty. These are feelings of exclusion, or feeling of inclusion occurring simultaneously with paradoxical exclusion, within the negative social connotations of Blackness. Interviewees reported stressful and anxious emotions present alongside working through an awareness that one’s self is viewed negatively in societal schemata in actuality and theoretically. In general, the ambivalent situatedness of suspected racism becomes crystallized in the hostility of unwanted attention in public spaces. One interviewee said: “…Germans have a habit of staring at people who look different, and they don’t feel ashamed about it.” (Interviewee 9). Commonly, they felt a pressure to engage habitually in intense public and private self-consciousness in spatial interactions. One participant said: “When I'm out in public, just whether it's on the U-Bahn, or in a cafe, anything, I feel like I'm hyper-aware of my ‘race’ more than anything else in Germany” (public self-consciousness), and “I feel like I have to, not that I do consciously all the time… I feel like I have to be a lot more palatable, and not come across as too threatening…” (private self-consciousness) (Interviewee 10). Interviewees described the intensity of emotions evoked (Interviewee 19). One said: “I feel like I am no one, I feel like I could just blend away in nothingness and life would continue for everyone around me” (Interviewee 3). Another said: “the most destructive feelings of depression happened to me because of racism…I was highly suicidal” (Interviewee 14).

### Paranoid ideation regarding general persecution

Racism causes a tendency to interpret the physical and socio-cultural environment as hostile, plus a propensity to assume that the likelihood that a racist agent will inflict harm is high. The interviewees revealed that the fear incited by hostility and the threat of racist attacks in spatial dynamics evoked a state of stress-based hypervigilance. They reported that to navigate public spaces in Germany, Berlin, and in their neighborhoods, they had to be on high alert for danger. One interviewee said: “you don't feel safe, you don't feel comfortable, you don't feel you can trust your environment, you have to sort of second-guess and analyze things that are said to you. You have to question; you have to question everything” (Interviewee 16). Interviewees spoke about a distinct sense of physical, psychological and emotional unsafety (Interviewee 18). This sense of unsafety impedes their willingness to travel around Germany and impacts the spaces they feel they can navigate on a more local, everyday level. Interviewees reported paranoid thought patterns that were, unlike traditional conceptualizations of paranoia, grounded in real-life experiences. One participant said: “I constantly felt like I was being watched. Also, because I was being watched” (Interviewee 9). As a result, chronic anticipation of emotional and physical harm and hurt manifests. They described going into a super alert state in public spaces to protect themselves and to avoid the surrealness and shock that accompanies racism (Interviewee 18).

### Self-questioning and self-esteem instability

Racism causes a reduction of self-concept clarity in the politics of exclusion resulting in fluctuations in positive and negative self-esteem, and in high and low self-esteem. Interviewees reported that racism caused insecurity in many different layers of selfhood. One interviewee said: “It sowed tons of insecurity in me about my job, my ability to do it, my value, my self-worth, am I smart, am I not smart, am I disciplined, is there anything I could have done differently” (Interviewee 3). As a result of the unpredictability and spontaneity of racist attacks, interviewees reported that their self-esteem is persistently vulnerable to challenge, which leads to fragile performances to attain validation. One interviewee said: “I was constantly trying to fit in, and pretend, and hide my African ways” (Interviewee 9). The realness of fatigue, despite the fact that, as one interviewee said, “[it is not] a scar that’s somewhere on your body that you can point out to people” (Interviewee 10), combined with society’s “nope, that is not real you’re delusional” (Interviewee 3), as another interviewee put it, forced a complicated oscillation between psychological hypervisibility and invisibility (Interviewee 7). One interviewee explained how this instability is more profound than a fluctuation, that the two states (positive or high self-esteem and negative or low self-esteem) coexist (Interviewee 6). As a result, this paradoxical mind state was experienced as confusion that distorted reality and self-perception, resulting in an incessant questioning of who they believe themselves to be and what they believe they are capable of achieving (Interviewee 14). Many stated struggling with self-doubt because the opportunities to be their authentic and natural selves were limited (Interviewees 3 and 8).

## Discussion

In this multi-method research approach, including the umbrella review and the qualitative interview study, we assessed meta-analytic data from 5 studies. The meta-analysis by Cantor-Graaee and Selten [46] reported the association between the developmental status of the country of origin and the incidence of schizophrenia. The meta-analysis by Bourque et al. [47] examined the incidence rate ratio for migrants concerning their areas of arrival. Kirkbride et al. [44] examined the incidence of schizophrenia, as well as other psychoses in England between 1959 and 2009, among other things, also regarding differences by region of origin. Nielssen et al. [43] examined the influence of the country of birth concerning inpatient hospitalization for treatment of psychosis compared to the native population from New South Wales, Australia. The data from this study was based on a comparison between census data from 2006 and patient registries from 2001 to 2010. Selten et al. [45] evaluated incidence studies related to migration and psychosis. More precisely, summary relative risk values were obtained for non-affective and affective psychotic disorders among migrants and their children (first- and second-generation). It was found, firstly, that migration from “developing” countries compared to migration from “developed” countries is more strongly associated with psychosis. Secondly, migration from the Caribbean and Sub-Saharan Africa is more strongly associated with psychosis than migration from the Middle East. Thirdly, there was no significant risk of psychosis associated with migration from North America, Asia, Northwest, Southern or Eastern Europe. Furthermore, while there was a significant risk of psychosis associated with migration to Great Britain, Scandinavia and the Netherlands, there was no significant risk associated with migration to Israel or Canada.

The umbrella review has several limitations. The robustness and comparability of the underlying data determine the robustness and comparability of the underlying meta-analyses and original investigations. This double layer of interpretation induces noise, limiting the detection of signals. Our umbrella analysis reveals high heterogeneity that limits the accuracy of the estimates. Reasons for this heterogeneity might be the different conceptualization of outcomes and risk factors in original articles and differences in eligibility criteria.

For an example, the included meta-analyses were screened for schizophrenia, first-episode psychosis or psychotic disorders in general. The majority did use the International Classification of Diseases (ICD-11) [48] or the Diagnostics and Statistical Manuel (DSM-5) [49] criteria for diagnosing affective or non-affective psychotic disorders. However, this could include F23.1 brief psychotic disorder (e.g., registry studies like the original article Leao et al. [50] that was included in the meta-analysis by Bourque et al. [47]), which could be an equivalent to a stress reaction in some cultural contexts inducing further possible confounds. Furthermore, the dichotomy between “developed” and “developing” is problematic and lacks the granularity of actual differences in a highly dimensional and dynamic system. However, classification could not be avoided in favor of good comprehensibility. We have taken care to mitigate these limitations with a comprehensive literature review, careful eligibility assessment, and a transparent quantitative assessment. Another limitation is that it would have been interesting to include refugee status as a moderating factor in the review; however, refugee status was not available in the literature and therefore could not be included in the analysis.

This umbrella review found that migration of Sub-Saharan African and Caribbean migrants is associated with the strongest risk of psychosis, which connects to theory and empirical data on the mental health consequences of being labeled as “inferior Others” and internalizing such racist ascriptions [51, 52]. Four racism-related vulnerability factors that impinge upon affect and cognition and potentially contribute to stress-associated mental burden/disorders emerged from the interviews: (1) a sense of differentness, (2) negative self-awareness, (3) paranoid ideation regarding general persecution, and (4) self-questioning and instability of self-esteem. Racism and the associated feelings of exclusion is posited as a social determinant of health that has a significant adverse impact on the mental health of migrants and further racialized groups [53]. Racism, according to previous stress and coping models, can become overpowering and impair abilities like meta-cognition and meta-affect [54]. When migrants are overwhelmed by racism, they can experience increased adverse mental health effects, such as depression, anxiety, and psychosis, making it difficult for them to participate productively in society, resulting in greater social costs to themselves [54]. Thus, the emotional distress caused by racism may contribute to stress-associated psychotic symptoms [55]. To better understand why Black migrants are at higher risk of psychosis, our qualitative interviews examined the relationship between racism and the manifestation of subthreshold psychosocial impairment. We found that experiences of racism and social exclusion contributed to specific feelings of imminent danger, fear and general anxiety, which may generalize into paranoid ideas of ubiquitous persecution.

These risk-associated and potentially dysfunctional cognitions may give rise to broad domains of atypical mental functioning, including intrusions and paranoid delusions, corresponding to an “affective route to psychosis” conceptualization [56]. In this context, a trans-diagnostic perspective alludes to the existence of vulnerability factors that facilitate a transition from pre-clinical psychotic symptomatology in the general population to clinical psychosis [57]. Our findings point to the confirmation of Frantz Fanon’s [58–60] argument for a “situational diagnosis”, a vocabulary that contextualizes the specific obstacles posed by racist discrimination, which shapes and organizes people’s “sense of security or insecurity” and “the dangers that threaten [them]” [61]. At the core of our conceptualization is that racism makes individuals and entire communities of people feel unsafe. Once developed, a network of perpetuating factors maintains these beliefs. Still, one crucial paradoxical oscillation precipitates: continued, repeated exposure to racism provides confirmatory evidence that they are unsafe, while avoidance of racism behaviors prevents disconfirmative evidence that they are safe. Treatment therefore should be high-level communication that reduces anxiety and engages honestly in the process of boundary setting and transcendence of racialized self- and community consciousness in racist and discriminatory societal contexts [62, 63]. This would be an iterative process centered on dealing with, and in turn transcending, racism-related unpleasant emotional experiences like external shame [64]. Future studies should examine the complex and bidirectional interaction between external shame (the feeling of being judged poorly by others), stigma, and racism in psychosis.

Racism is widely posited in the literature as potentially causing traumatic stress [65–68], which in turn strongly increases the likelihood of developing post-traumatic stress disorder (PTSD) and psychosis [69]. For Black people and People of Colour, racism can be traced to multiple dimensions of interpersonal and impersonal (vicarious) trauma—from murder to grievous bodily harm to police brutality, and genocide [70]—many of which are structural, with broad implications for health [71]. Our qualitative study reports broad levels of racist exclusion and traumatization, which adds to the growing body of evidence that the emotional abuse of racism in all of its manifestations is a significant source of affective distress for Black people and People of Color. Rather than emphasizing psychiatric diagnoses, our qualitative study was designed to contextualize a mental health disparity by attempting to understand anomalies of experience as emanating from and anchored in social contexts of interactions [72]. This social cognitive approach among Black people living in Berlin aims to better understand how they make sense of their experiences of racism and how subclinical psychosis symptomatology may manifest in response to racism in everyday life. Altogether, our findings can help to understand how psychosis may emerge over time in the context of an accumulation of racist encounters [5, 73]. There is substantial evidence that fear and anxiety may induce paranoid thinking in people with PTSD, similar outcomes have also been found in people with psychosis. The meta-analysis by Tully et al. [74] on how persons with psychosis manage their paranoid ideation regarding general persecution, focuses on methods such as avoidance, escape and resistance, which they label as safety-seeking behaviors.

Regarding potential interventions, a paradigm shift from cross-cultural competency—defined as a set of psychosocial skills such as empathy and flexibility that facilitate everyday clinical encounters with diverse groups [75]—to structural competency entails recognizing the structural factors that cause and perpetuate mental illness. As well as the structural factors that influence both the patient and clinician in the texture of clinical interactions, and the structural factors that influence the cultural formulations in terms of diagnosis and treatment [76]. Furthermore, it requires adequate reflection on the ethnocentric racism embedded in Western mental health science and what this means in service provision for non-*white* racialized/minority patients [77, 78]. Fernando [29] highlights overdiagnosis (the mislabeling of real racist discrimination) explicitly and distinguishes it from a genuine increased risk (the stress due to racist experiences). In future research, a complex examination is needed to investigate the extent of conceptual overlap between patterns of racism-induced thoughts (grounded in reality) and racism-induced paranoid ideation (not grounded in reality) in racialized populations.

A large section of the research on the mental health consequences of racism has focused on stress [79, 80]. The induction of attenuated psychotic symptomatology as a stress-induced mental state has received little attention in the context of measuring the association between racism exposure and adverse mental health. Given the relevance of the topic and to avoid it being neglected, further evidence is needed to ascertain whether greater levels of racism are associated with greater severity of attenuated symptoms [55]. Further to this, we recommend additional research to ascertain the association between attenuated symptomatology and different classes of racism (avoidant, hostile, aversive-hostile), in addition to different forms of racism (structural, institutional, interpersonal and vicarious) [81]. To build a more complete etiological picture of psychosis from a social perspective situated within institutional racism theory, country-specific racism item parameters are needed [29]. Specifically, integrating the complexities of various forms and domains of racism—while taking into account local and regional psychosocial stressors—into models of psychosis operationalized as the presence of subclinical positive symptoms as evaluated with the Prodromal Questionnaire [82], for example, may help to build a more sophisticated etiological picture of psychosis in vulnerable populations.

## Supplementary Information

Below is the link to the electronic supplementary material.

Supplementary file 1 (PDF 64 KB)

Supplementary file 2 (PDF 84 KB)

Supplementary file 3 (R 3 KB)

Supplementary file 4 (PDF 60 KB)

Supplementary file 5 (PDF 67 KB)

Supplementary file 6 (PDF 113 KB)

Supplementary file 7 (PDF 114 KB)
