# Supplementary material for: Racism and psychosis: an umbrella review and qualitative analysis of the mental health consequences of racism
Source: Eur Arch Psychiatry Clin Neurosci. 2022 Aug 24;273(5):1009–22. doi: 10.1007/s00406-022-01468-8 (PMC9400567; doi:10.1007/s00406-022-01468-8)
Supplement: Supplementary file 1 — (PDF 64 KB) [file 406_2022_1468_MOESM1_ESM.pdf]

Supplementary material 1. Data extraction of the included meta-analyses

pooled	Studie	FGM SGM	Riskfactor	Impact	Diagnosis	k	N1cases	N2controls	N3 = sample size	Random_effects_measure	value	lower	upper	p	Common_effects_measure	valueD
4	Selten et al., 2	FGM, SGM	"developed" c	Mig	NAP	19				RR	1.66	1.49	1.84		d	0.28
4	Cantor-Graae	FGM, SGM	"developed" c	Mig	SCZ	11	980	14868	15848	RR	2.3	1.7	3.1		d	0.46
5	Selten et al., 2	FGM, SGM	"developing c	Mig	NAP	30				RR	2.54	2.26	2.86	<0,001	d	0.51
5	Cantor-Graae	FGM, SGM	"developing c	Mig	SCZ	35	2140	25904	28044	RR	3.2	2.8	3.9		d	0.64
6	Selten et al., 2	FGM, SGM	to Great Brita	Mig	NAP	18	2025	6528	8553	RR	2.69	2.2	3.28		d	0.55
6	Bourque et al	FGM	to Great Brita	Mig	PD	24	1291	5843	7134	IRR	2.8	2.2	3.5		d	0.57
6	Bourque et al	SGM	to Great Brita	Mig	PD	9	148	200	348	IRR	3.7	2.1	6.6		d	0.72
7	Selten et al., 2	FGM, SGM	to Scandinavi	Mig	NAP	11	6651	37043	43694	RR	1.89	1.79	2		d	0.35
7	Bourque et al	FGM	to Scandinavi	Mig	PD	15	1919	14135	16054	IRR	2.3	1.9	2.7		d	0.46
7	Bourque et al	SGM	to Scandinavi	Mig	PD	8	2518	23843	26361	IRR	1.8	1.6	2		d	0.32
8	Selten et al., 2	FGM, SGM	to the Nether	Mig	NAP	4	2298	13198	15496	RR	2.98	2.43	3.66		d	0.60
8	Bourque et al	FGM	to the Nether	Mig	PD	10	1091	11780	12871	IRR	2.5	2	3.2		d	0.51
8	Bourque et al	SGM	to the Nether	Mig	PD	6	48	79	127	IRR	3	2.1	4.4		d	0.61
9	Selten et al., 2	FGM, SGM	to Israel	Mig	NAP	3	3252	1406	4658	RR	1.22	0.99	1.5		d	0.11
9	Bourque et al	FGM	to Israel	Mig	PD	7	284	46	330	IRR	1.5	1.1	2.1		d	0.22
9	Bourque et al	SGM	to Israel	Mig	PD	5	1801	238	2039	IRR	1.1	0.9	1.3		d	0.05
np	Selten et al., 2	FGM, SGM	to Southern E	Mig	NAP	4	293	754	1047	RR	2.79	1.94	4.01		d	0.57
np	Selten et al., 2	FGM, SGM	to Canada	Mig	NAP	2	2853	21218	24071	RR	1.21	0.85	1.74		d	0.11
10	Kirkbride et a	FGM, SGM	from the Cari	Mig	SCZ	5	271	403	674	RR	5.6	3.4	9.2		d	0.95
10	Bourque et al	FGM	from the Cari	Mig	PD	12	1487	1625	18012	RR	3.9	3.4	4.6		d	0.75
10	Bourque et al	SGM	from the Cari	Mig	PD	7	224	10048	10272	RR	5.8	3.5	7.4		d	0.97
13	Selten et al., 2	FGM, SGM	from North A	Mig	NAP	4				RR	2.88	1.85	4.49		d	0.58

Supplementary material 1. Data extraction of the included meta-analyses

13	Nielssen et al	FGM	from North A	Mig	PD	10				OR	1.02	0.76	1.37		d	0.01
11	Kirkbride et a	FGM, SGM	from Sub-Sah	Mig	SCZ	5	271	403	674	RR	4.7	3.3	6.8		d	0.85
11	Bourque et al	FGM	from Sub-Sah	Mig	PD	6	224	10048	10272	RR	4.3	2.8	6.8		d	0.80
11	Nielssen et al	FGM	from Sub-Sah	Mig	PD	10				OR	1.04	0.87	1.25		d	0.02
11	Selten et al., 2	FGM, SGM	from Sub-Sah	Mig	NAP	13				RR	2.99	2.3	3.89		d	0.60
np	Bourque et al	FGM	from the Mid	Mig	PD	5	121	9738	9859	RR	2.3	1.4	4		d	0.46
np	Bourque et al	SGM	from the Mid	Mig	PD	2	36	79	115	RR	2.3	1.4	4		d	0.46
12	Kirkbride et a	FGM, SGM	from Asia	Mig	SCZ	3	259	165	424	RR	2.4	1.3	4.5		d	0.48
12	Bourque et al	FGM	from Asia	Mig	PD	7	326	13711	14037	RR	1.7	1.3	2.3		d	0.29
12	Bourque et al	SGM	from Asia	Mig	PD	2	21	108	129	RR	1.3	0.8	2.1		d	0.14
12	Nielssen et al	FGM	from Asia	Mig	PD	10				OR	0.77	0.55	1.09		d	-0.14
12	Nielssen et al	FGM	from Asia	Mig	PD	10				OR	0.82	0.63	1.08		d	-0.11
12	Nielssen et al	FGM	from Asia	Mig	PD	10				OR	0.93	0.67	1.3		d	-0.04
12	Selten et al., 2	FGM, SGM	from Asia	Mig	NAP	7				RR	1.65	1.31	2.07		d	0.28
12	Selten et al., 2	FGM, SGM	from Asia	Mig	NAP	15				RR	1.52	1.22	1.89		d	0.23
np	Nielssen et al	FGM	from New Zea	Mig	PD	10				OR	1.16	0.51	2.65		d	0.08
np	Nielssen et al	FGM	from Ozeania	Mig	PD	10				OR	1.65	1.16	2.35		d	0.28
14	Nielssen et al	FGM NwEU	from North-W	Mig	PD	10				OR	0.81	0.61	1.07		d	-0.12
14	Selten et al., 2	FGM, SGM	from North-W	Mig	NAP	13				RR	1.62	1.41	1.86		d	0.27
15	Selten et al., 2	FGM, SGM	from South- c	Mig	NAP	7				RR	1.93	1.38	2.7		d	0.36
15	Nielssen et al	FGM	from South- c	Mig	PD	10				OR	1.25	1.03	1.51		d	0.12
np	Nielssen et al	FGM	from the US	Mig	PD	10				OR	1.21	0.89	1.64		d	0.11

Supplementary material 1. Data extraction of the included meta-analyses

lowerD	upperD	I2	p(I2)	Q	p(Q)
0.22	0.34	97.1			
0.29	0.62				
0.45	0.58	97.6			
0.57	0.75				
0.43	0.65	98.7			
0.43	0.69	93		237.2	<0,01
0.41	1.04	87.6		64.5	<0,01
0.32	0.38	93			
0.35	0.55	92.4		185	<0,01
0.26	0.38	84.8		46	<0,01
0.49	0.72	84.5			
0.38	0.64	85.3		61.2	<0,01
0.41	0.82	30.7		7.2	
-0.01	0.22	95.5			
0.05	0.41	51.9		12.5	<0,1
-0.06	0.14	68.8		12.8	<0,05
0.37	0.77	83.6			
-0.09	0.31	91.2			
0.67	1.22	77			
0.67	0.84	74.2		42.6	<0,01
0.69	1.10	77.2		26.3	<0,01
0.34	0.83	68.7			

Supplementary material 1. Data extraction of the included meta-analyses

-0.15	0.17				
0.66	1.06	47			
0.57	1.06	86.9		38.1	<0,01
-0.08	0.12				
0.46	0.75	91.6			
0.19	0.76	87.5		31.9	<0,01
0.19	0.76	65.8		2.93	<0,01
0.14	0.83	42			
0.14	0.46	81.4		32.2	<0,01
-0.12	0.41	0		0.06	<0,01
-0.33	0.05				
-0.25	0.04				
-0.22	0.14				
0.15	0.40	79.6			
0.11	0.35	89.2			
-0.37	0.54				
0.08	0.47				
-0.27	0.04				
0.19	0.34	98			
0.18	0.55	93.3			
0.02	0.23				
-0.06	0.27				
