# Supplementary material for: Racism and psychosis: an umbrella review and qualitative analysis of the mental health consequences of racism
Source: Eur Arch Psychiatry Clin Neurosci. 2022 Aug 24;273(5):1009–22. doi: 10.1007/s00406-022-01468-8 (PMC9400567; doi:10.1007/s00406-022-01468-8)
Supplement: Supplementary file 2 — (PDF 84 KB) [file 406_2022_1468_MOESM2_ESM.pdf]

Supplementary material 2. Quality assessment: AMSTAR index (2007)(1)

Author	a priori design?	Duplicate study selection & data extraction?	Performance of comprehensive literature search?	Status of publication used as inclusion criteria?	List of studies included and excluded provided?	Characteristics of included studies provided?	Scientific quality of included studies assessed, documented?	Scientific quality of included studies used appropriately for formulating conclusions?	Methods used to combine studies appropriate?	Assessment of likelihood of publication bias?	Conflict of interest stated?	AMSTAR rating
Selten et al. 2020 (2)	y	y	y	y (published peer review only)	n	y	y	y	y	y	y	9/11
Cantor-Graae et al. 2005 (3)	y	y	y	y (published peer review only)	n	y	n	y	y	y	n	8/11
Bourque et al. 2011 (4)	y!	y	y	y (published peer review only)	n	y	y	y	y	y	y	10/11
Kirkbride et al 2012 (5)	y	y	y	y + unpublished	n	y	y	y	y	y	y	10/11

1. Shea BJ, Grimshaw JM, Wells GA, Boers M, Andersson N, Hamel C, et al. (2007). Development of AMSTAR: a measurement tool to assess the methodological quality of systematic reviews. *BMC Medical Research Methodology*. 7(1):10.
2. Selten JP, van der Ven E, Termorshuizen F. (2020). Migration and psychosis: a meta-analysis of incidence studies. *Psychol Med*. 2020;50(2):303-13.
3. Cantor-Graae E, Selten JP. (2005). Schizophrenia and migration: a meta-analysis and review. Am J Psychiatry. 162(1):12-24.
4. Bourque TJ, Hoy K, Shannon C. A (2014). A systematic review and meta-analysis of the ethnic density effect in psychotic disorders. *Soc Psychiatry Psychiatr Epidemiol.* 2014;49(4):519-29.
5. Kirkbride JB, Errazuriz A, Croudace TJ, Morgan C, Jackson D, Boydell J, et al. (2012). Incidence of schizophrenia and other psychoses in England, 1950-2009: a systematic review and meta-analyses. *PLoS One.*7(3):e31660.

N. B. The included meta-analysis by Nielssen et al [37] was performed upon data from registries which meant that many of the points of the AMSTAR ratings could not be answered.
