# Supplementary material for: Racism and psychosis: an umbrella review and qualitative analysis of the mental health consequences of racism
Source: Eur Arch Psychiatry Clin Neurosci. 2022 Aug 24;273(5):1009–22. doi: 10.1007/s00406-022-01468-8 (PMC9400567; doi:10.1007/s00406-022-01468-8)
Supplement: Supplementary file 4 — (PDF 60 KB) [file 406_2022_1468_MOESM4_ESM.pdf]

Supplementary material 4. Semi-structured interview topic guide

---

1. Could you tell me a little bit about yourself?
    - How would describe yourself based on your current life?
    - Where would you consider as home?
2. How do you feel about Germany?
3. Are you able to feel at home in Germany or is there a sense of marginality?
4. To what extent do you feel accepted and part of German society?
5. In what circumstances have you felt that social exclusion is a problem?
6. What challenges or barriers have complicated your sense of belonging?
7. What ways have you experienced or are you experiencing less access to opportunities, for example for political and social participation, because of your background?
8. What in your view does the term integration refer to, and is it something positive or negative?
9. What is the relationship between stress, mental health and migration?
10. Have you received support from family and friends throughout it all?
11. How do the people in your life talk about racism?
    - What is said about racism against people of African descent?
    - Are there conflicting views?
12. To what extent do you agree that racism is a highly stressful challenge for people of African descent in Berlin?
13. In what ways can experiencing racism have profound effects on people's mental health?
14. Can you tell me a little bit about how racism has impacted your mental health?
    - Were you able to get support from mental health services?
15. How do people in your life talk about sexism?
    - What do they say about sexism?
    - Are there conflicting views?
16. In your opinion, in comparison to African men, is the impact of racism different for women of African descent?
17. To what extent do you agree that sexism is a stress factor imposed on women that can affect their mental health?
18. What are some forms of sexism you have experienced?
19. How has discrimination based on the fact that you are a women (or a man) impacted your experiences in Germany?
20. Can you tell a little bit about how sexism has impacted your mental health?
    - Were you able to get support from mental health services?
21. Do you consider yourself a feminist?
    - What does feminism mean to you?
22. What does intersectionality mean to you?
    - What does it look like?
    - What its impact across Global North-Global South divides?
23. What is privilege?
    -what are some personal experiences you've had in terms of dealing with or noticing privilege?
    - why is it important that we recognise privilege in our lives?
24. What is solidarity in the context of intersectional feminism?
25. How can we become more aware of opportunities to challenge and renegotiate power?
26. What does resilience-building look like and feel like to you?
27. What mental health inequalities exist in regards to women (or men) of African descent?
28. Do you think women (or men) of African descent would want to get involved in this type of research?
29. What types of challenges need to be addressed in order to make it easier for women (or men) of African descent to participate in this type of research?
30. Do you feel that mental health services are safe for women (or men) of African descent to utilise?
