# Supplementary material for: Racism and psychosis: an umbrella review and qualitative analysis of the mental health consequences of racism
Source: Eur Arch Psychiatry Clin Neurosci. 2022 Aug 24;273(5):1009–22. doi: 10.1007/s00406-022-01468-8 (PMC9400567; doi:10.1007/s00406-022-01468-8)
Supplement: Supplementary file 6 — (PDF 113 KB) [file 406_2022_1468_MOESM6_ESM.pdf]

Supplementary material 6. Coding structure

1. A sense of differentness

3rd level codes	2nd level codes	1st level codes	Quotes
The Black body is made into an ontological problem in relation to the white gaze	The Black (female) body within the context of whiteness	The objectification of women and exotification of women of colour	"First of all the feedback from the outside that I look different and the exotification in either people telling me that I'm different, behave differently, I do different things" (Interviewee 1).
			"When I enter certain situations the possible threat of being harassed or being commented on because I'm female and because I am black and because I know that my body is seen and valued differently, especially by white men, it's stressful and it starts when you again enter public transport" (Interviewee 7).
			"When I'm home in Namibia, I'm normal and normal-looking. And I feel normal. I don't think that this or that is necessarily too much, because it's just how my body naturally is. And here, the attention that I've gotten from white men has really been disturbing and creepy. I think the idea that certain people here have about what black women are like, and that always seems to be something sexual. Really. There's sometimes this feeling of like, "that's what we're here for, or?" (Interviewee 10).
	The white entitlement zone saturated with value-laden meanings in the politics of space	The invasion of personal space in public spaces	"Racism is like a heavy, like a like a dark cloud that always follows you. It starts when we use public transport. There's always a chance that you will meet a racist person, and as a woman people, even white women, approach you differently and it starts by staring or being photographed without your consent. People making fun of you" (Interviewee 7)
			"As I leave the house I see how people stare at me when people look at my hair and then suddenly touch their own hair and then in worst cases like often when I went outside in the summer people definitely started taking pictures of me" (Interviewee 14).
	The social construction of whiteness in social spaces	The forced situatedness of Black people as deviating from the white normal	"You're always different everywhere. At work, at school, at church, you're different. You have different realities from other people which is important. You have realities that you have to explain and everybody else doesn't have to explain their reality" (Interviewee 3).<br><br>"I have a lot of privilege, I have quite a lot of privilege because I do have an educated background, I was very well educated in Zim, my education was Eurocentric so I speak fluent English and I know how to use a knife and fork, I know which one is the dessert spoon just because

			of the way I grew up. My proximity to Europeanness is a little more than another African. I have all that privilege and I acknowledge that it is a little easier for me to be understood than someone else. And I know this, and I have had so many conversations about this, but even then it's still a lot of hard work for me to blend in. Just to blend in and have us all be on the same page at school or work, it is still a lot of work" (Interviewee 3).
			"There is racism here. And racism is not necessarily that someone says something bad or do something bad, but it's just like the attitudes people might have, or beliefs that they might have: these kinds of limitations. And just the fact that from appearance, when I go somewhere, usually I'm the only African person there. So I'm immediately different from everybody else. It's no limitation to be African, but it is a limitation in this situation" (Interview 15).<br><br>"This system itself is designed to give stress, especially to African people. Because you know an African person can not blend in, as much as you might want to try to blend in you won't be able to blend into the culture, just because you, we, other African people have a different body type. So that's going to always give stress because the culture is designed that way" (Interviewee 15).
			"I look different. So sometimes when I speak German, then people are more accepting but because I look different and when people don't know me, I don't feel accepted in any way. People would feel the need to explain things to you in a supermarket or weird things happen like when I go on for a walk, you might be avoiding something on the track and someone comes with a bicycle and they feel like explaining something to you, like 'hey, you guys that are not from here you don't know how things work' or something. I don't know. This is weird. White people wanting to explain things to you" (Interviewee 2).
Negative affective consequences of being viewed as a deviation from the white norm	Emotional alienation within the social connotations of Blackness	The internalisation of objectification and exotification	"Basically, people's perceptions of my looks and my behaviour have made me feel like I don't belong because otherwise I probably wouldn't even have noticed as a kid" (Interviewee 1).
		The internalisation of white normativity	"Racism has definitely affected me from day one, it started from kindergarten and it was subconsciously processed where I took over these white ideas of blackness and grew up hating myself or thinking that I am weird and different" (Interviewee 7).
			"I supposed that it was normal that I was feeling like this. I felt that being an African in Europe I felt that I had to adjust to come here. I felt the anxiety and the fear that I was feeling was just the process of becoming a much more civilised, African woman" (Interviewee 9).
		The perceived task difficulty of incongruity adaptation	"I am different, I am incredibly different. I have had to seek out relationships with Black people… I am just saying that to say that the consequence of my existence is that I am different…a difficult thing for anyone to adapt to, is always being different wherever you go and unfortunately it is not like everyone is different. You are the different one" (Interviewee 3).

2. Negative self-awareness

3rd level codes	2nd level codes	1st level codes	Quotes
Feelings of inadequacy (not being good enough) and uncertainty in the social location of Blackness	Insecurity	Direct, interpersonal experiences of the objectification of foreignness	“Being watched and judged which is not an easy dynamic to deal with. It can make you feel very insecure and it affects you if you care about what other people think and say and how they might treat you” (Interviewee 1).
			“I constantly felt like I was being watched. Also, because I was being watched. Germans have a habit of staring at people who look different and they don’t feel ashamed about it. So because of this constant being watched I kind of developed anxiety, like going out of the house, going to the train station, going into the shop” (Interviewee 9).
			“I surely cannot be myself. When I dress traditionally, like in my Facebook profile, for example, there are different reactions to that - some are laughing, some are just staring at me like in a zoo” (Interview 11).
			“As I leave the house people are staring at me, people make stupid remarks and when I was younger and I went into clubs either people attacked my hair or saw me as something exotic like this” (Transcript 14)
			“I felt it a lot in social spaces in the sense of l going to the grocery store. You're constantly watched and monitored and asked questions and they're not doing that to Germans or anyone else. And, and it makes me feel very uncomfortable...I know if I looked a certain way that wouldn't have happened. Sometimes I actually get palpitations going to the store. Just because I feel the trauma I've received in the past, when I am almost like terrorized in the grocery store” (Interviewee 18).
		The internalisation of foreigner objectification (self-objectification)	“I don't know if it's because I'm black or because I'm African. Someone might say one thing, and then I will carry it with me and the lack of confidence that I might have will make me less likely to speak up. And this affects me in going for things” (Interviewee 2).
			“I definitely feel as though when I step out of my home, I'm very, very, very, very much aware of my race every single day. When I'm out in public, just whether it's on the Ubahn, or in a cafe, anything, I feel like I'm, I'm hyper-aware of my race, more than anything else in Germany. That hyper-awareness is super stressful, because a lot of the times I feel like I have to, not that I do consciously all the time, but I do feel that I have to sort of make the people around me comfortable” (Interviewee 10)
			“It sowed tons of insecurity in me about my job, my ability to do it, my value, my self worth, am I smart, am I not smart, am I disciplined, is there anything I could have done differently…. it sows seeds of insecurity and some kind of pain” (Interviewee 3).

Feelings of exclusion or inclusion simultaneously with paradoxical exclusion within the social connotations of Blackness	Emotional alienation	Otherness as a form of intersubjective social exclusion	“I'm always constantly reminded that I'm not a full hundred percent part of the society or not seen as as a full part of the society because I'm black and I have features that don't fall into the racist or stereotypical concept of a German. I'm not white, I don't have straight hair, I don't have light eye colour…growing up here has always been experiencing being othered and alienated on an almost daily basis, and it does something to you. It did something to me” (Interviewee 7).
			“No matter where you are, and where white people are, you somehow felt always this tension and though there no law that says you're not coming in here, yes in the immigration laws there is, but as soon as I'm here there's no law officially but I feel it everywhere I go” (Interviewee 14).
		Blackness, the target of white toleration	“I feel tolerated. and if you are asking to what extent do I feel tolerated, maybe to like fifty percent…I feel tolerated. i don’t know if I feel accepted at all… my short answer to you is no, the society does not accept me, it tolerates me” (Interviewee 3).
			“I think I'm not accepted. I think I'm more tolerated. I'm tolerated…I don't feel like I have been treated in a way that would have made it possible for me to feel at home. To feel home would have been for them to be much stricter with kids, to be like “no, you don't use the N-word”, “no, you don't make fun about her hair”, “you don't say these kinds of things about her skin”, it would have needed people, like authorities, to be stricter, to educate white kids on how to treat a black kid correctly. For that black kid to feel welcome. So for me, no, I don't feel accepted. I just feel tolerated” (Interviewee 17).
			“I don't think I've ever felt accepted. I think I'm tolerated. Because, you know, I was able to provide the right paperwork in order to get a visa and I have reason to stay. So I've never felt accepted. I've only been tolerated because I've proven that I have reason to be here” (Interviewee 18).

3. **<u>Paranoid ideation regarding general persecution</u>**

3rd level codes	2nd level codes	1st level codes	Quotes
A tendency to interpret the physical and cultural environment as hostile	The paradox of (in)visibility and hypervisibility in spatial dynamics	The feeling of being watched	“Constantly being watched and judged which is not an easy dynamic to deal with. It can make you feel very insecure and it affects you if you care about what other people think and say and how they might treat you” (Interviewee 1).
			“I don't want to be chased by Nazis and I don't want to get beaten up. That's one thing, but I want to have a vacation where I have my peace of mind and that includes people not staring at me or starting to whisper something when I pass them or looking all the time” (Interviewee 7).

			“I constantly felt like I was being watched. Also, because I was being watched. Germans have a habit of staring at people who look different and they don’t feel ashamed about it. So because of this constant being watched I kind of developed anxiety, like going out of the house, going to the train station, going into the shop” (Interviewee 9).
			“As soon as I leave the house I see how people stare at me” (Interviewee 14).
			“You're constantly watched and monitored and asked questions and they're not doing that to Germans or anyone else. And, and it makes me feel very uncomfortable. Because, for example, I'm just trying to get bread” (Interviewee 18).
			“When I walk through the street, I'm aware that a lot of people, they look at me, and they automatically assume that I must be an African immigrant” (Interviewee 19).
			“I felt super uncomfortable and super stared at all the time.” (Interviewee 10).
		The feeling of being targeted	“It's not the most diverse place in the world, there's a lot less diversity here than what I'm used to. And I feel a lot more seen, but not necessarily in a good way. I feel like I stand out more. And there's been a lot of hostility at times, since a lot of circles that I'm in, tend to be white” (Interviewee 10).
			“I've experienced a lot of hostile sexism. Yeah, I think it's been very hostile towards me because I'm black. And because I'm a woman. A lot of people already have assumptions and have stereotypes towards you like, and I think the minute you walk into a room, your politics is already decided for you, how you're supposed to be is really decided for you. So I think it's been very, very hostile. And I'm always seen as inferior, also for the fact that I'm, you know, a black woman” (Interviewee 18).
			“No matter where you are, and where white people are, you somehow felt always this tension and though there no law that says you're not coming in here, yes in the immigration laws there is, but as soon as I'm here there's no law officially but I feel it everywhere I go” (Interviewee 14).
			“It brings stress because of the way the culture is made. The culture is designed to give people like myself some kind of anxiety or fear…it is always a design of the culture… you're never quite sure who you're meeting or who you come up against, you don’t know if they're gonna attack you…I try not to stress myself as much as possible, but it's actually built into the culture.” (Interviewee 15).

A propensity to assume that the likelihood that a racist agent will inflict harm is high	The feeling of unsafety	Perceived threat	“I think it's this feeling of instability, this feeling of unsafety this feeling of being unwanted, rejected, I think all of these things have serious consequences on people's mental health and, and just people's level of normality.. I think if you constantly live in a state of I'm not safe, I'm not wanted, that that can really affect you” (Interviewee 18).
			“…being othered and alienated on an almost daily basis, it does something to you. It did something to me. I don't feel safe in any part of Germany. I don't travel to certain parts of Germany because I know I won't be safe there, I cannot have a good time as maybe white people or people who are read as white can have” (Interviewee 7).<br><br>“I would call Berlin my home because of the people, because of the spaces that I can navigate, that I can enter. But as a whole Germany is a different thing. I don't, I can't call a place my home where I don't feel safe and by safe I mean physically safe, psychologically safe and where I don't have to experience all of these kinds of microaggressions that increase if I leave Berlin” (Interviewee 7).
			“I used to be super adventurous and it's coming back, but definitely the worst phases are always the phases is where you get more careful and I think this is a part of mental health. That's really important because feeling confidence and able to do stuff and feel safe doing it is the foundation for many thing in life. This is one of the ways also that people are robbed of their opportunities.” (Interviewee 8).
			“You’re anxiety-driven, you're constantly, you don't feel safe, you don't feel comfortable, you don't feel you can trust your environment, you have to sort of second guess and analyze things that are said to you. You have to question, you have to question everything. And it's a process that's very crazy” (Interviewee 16).<br><br>“…you're constantly, you don't feel safe, you don't feel comfortable, you don't feel you can trust your environment, you have to sort of second guess and analyse things that are said to you. You have to question, you have to question everything” (Interviewee 16).
		Threat contol-override	“I feel like I have to be a lot more palatable, and not come across as too threatening. Or if I'm just stepping out from my place, and I need to rush to the grocery store and I don't look very put together. I try to not look very masculine for example, when I'm outside or when I go into super white spaces. It's very stressful, and it's anxiety-inducing. But I also feel like if I don't do it, I'll be a lot more uncomfortable, I guess. It feels like a lot of emotional labour that I have to do for people that I don't even know, just totally strangers” (Interviewee 10).
			“...it was just this anxiety and fear of being outside, of behaving in a particular way that is too African. I was constantly trying to fit in, and pretend, and hide my African ways. And sometimes it would come out because you can’t hide these types of things. Just in the middle of code-switching, as I'm talking, I find myself talking in my African accent. It was mainly just the anxiety and the constant feeling of having to be somebody else when I am in particular crowds and then be myself when I'm alone” (Interviewee 9).

			“I'm not scared all the time and I'm not angry about it all the time but I know that there is a part in myself that is always ready to get into standby mode. Going back to my set of ‘what can I do to protect myself when I enter certain situations’, when I enter certain situations the possible threat of being harassed or being commented on because I'm female and because I am black and because I know that my body is seen and valued differently, especially by white men, it's stressful and it starts when you again enter public transport - ‘who I am sitting next to?’ - this is a very important choice and looking at other people - ‘is someone looking at me in a strange way?’ ‘Is someone approaching me?’ (Interviewee 7).

4. **<u>Self-questioning and self-esteem instability</u>**

3 levels codes	2nd level codes	1st level codes	Quotes
Reduction of self-concept clarity in the politics of exclusion	Mental habit of questioning own worth	systematic devaluation of merits and contributions	“if I accomplish something, it's cute, it is not an accomplishment. The responses are like, it's adorable that she managed to do all that despite...despite what? You know. There is a certain derogatory way in which you are viewed, where it is like, this lesser being actually pulled it off. And that to me is exhausting and to me makes me question myself” (Interviewee 3).
			“Even if they don't say it out loud, they don’t believe it, they don’t believe that you are as capable or fast as the guy sitting next to you.Something in the body language and the words they use reinforces that and it makes me question, I have to do extra work mentally and emotionally just to be able to believe in myself and my ability to work in tech and exist in this space and that is exhausting as well” (Interviewee 3).
			“Many of my insecurities and my anxiety and my general sadness and not feeling comfortable about myself was also the product of racism…I had my issues with my blackness, but at the other hand, I always loved my blackness but at some point, I realized that society made it hard for me to stand by this decision…I will always be surrounded by a system that tries to minimize me or make me invisible because I'm black and because I'm a woman, both of it. And yet the way racism affected me, it made me always question what I thought I can be and what I thought I want to be” (Interviewee 7).
		Negative self-image	“I’d come home every day and be like, ‘what's wrong with my character?...and we'll try and figure out what was wrong” (Interviewee 8).
			“It makes you question yourself. it creates this distorted view of who you are and how well you’re doing and how hard you need to work. Then that distorted view creates stress because you are doing everything you should be doing but you're not getting the results that you should be getting because of your race. So we are stressed about it. And whether you are aware that it is being done because of your race, or you’re unaware that it is being done because of your race, it is stressful. it is also stressful to be excluded” (Interviewee 3).
	Fragile performances	Denial of reality defence	“It makes you feel less valid and also makes you feel as if you imagined those things. For me, I always have a question about if it is real what I

	to attain validation from within self and from others	mechanism	am experiencing or is it there which has the impact that I was not sure about myself, about my self worth, my self-esteem, validation, things like that. I was always seeking things, and in different ways, just to feel valued” (Interviewee 4).
		Negative self-image	“I have to prove myself in order to be afforded my humanity. This has everything to do with being Black. If you’re white and straight and whatever, male, then you are a human being even if you murder someone, then the media will start by describing you as a human being. You’re a human being first. Whereas if you are Black, you are everything but a human being, you are a statistic before you’re a person, you are a representative of your nation before you’re a human being. I have to earn it, I have to be eloquent and I have to be efficient and I have to be punctual and I have to be smart and I have to be pleasant in order for me to be embraced by my co-workers. I can’t just be. And maybe embraced wasn’t the right term, but in order for them to see me and be like, oh she’s African but she is just like the rest of us, I have to put the work in first. I have to put in work to be understood and I have a lot of privilege.” (Interviewee 3).
			“Never in my entire life have I experienced, I didn’t even know what it was, having sweaty moments in winter or panicking about the tiniest things like just going outside and taking the train….during this time, I felt like I was trying so hard to be or appear German so that people would stop looking at me or so that they wouldn’t behave strangely around me…It kind of made me not like the place because I was so focused on how I was being perceived and I was so desperate to integrate and to be part of the German society, as an intellectual… it was just this anxiety and fear of being outside, of behaving in a particular way that is too African. I was constantly trying to fit in, pretend, and hide my African ways” (Interviewee 9).
Fluctuations from positive-negative self-esteem, and from high to low	Psychological invisibility within the contradiction of acceptance	The ambiguity of vulnerability	“I would describe myself as a very strong yet vulnerable person. If I should describe myself I would say I'm strong, but at the same time very vulnerable…I can also be a very insecure person dealing with anxiety and self-consciousness. And this is still a problem that I have to tackle but at the same time I am also quite confident about myself…Sometimes very ambiguous. I can be self confident, and in the next moment, I can be very self-conscious of things, but it depends.” (Interviewee 7).
			“I do relate The Strong Black Woman up to a certain point but right now I feel like an extremely extremely weak Black woman. For me it is totally fine for them to co-exist, one doesn’t diminish the other” (Interviewee 6).
			“...I had my issues with my blackness, but on the other hand, I always loved my blackness but at some point, I realised that society made it hard for me to stand by this decision. And of course, as a teenager, you just want to fit in and not really stand out. But I was always made the person who stands out, although I didn't want to…they distract you from becoming yourself because you always constantly have to deal with the effects that these discriminations have with you” (Interviewee 7).
